# Supplementation with *Lactobacillus plantarum* WCFS1 Prevents Decline of Mucus Barrier in Colon of Accelerated Aging *Ercc1^−/Δ7^* Mice

**DOI:** 10.3389/fimmu.2016.00408

**Published:** 2016-10-07

**Authors:** Adriaan A. van Beek, Bruno Sovran, Floor Hugenholtz, Ben Meijer, Joanne A. Hoogerland, Violeta Mihailova, Corine van der Ploeg, Clara Belzer, Mark V. Boekschoten, Jan H. J. Hoeijmakers, Wilbert P. Vermeij, Paul de Vos, Jerry M. Wells, Pieter J. M. Leenen, Claudio Nicoletti, Rudi W. Hendriks, Huub F. J. Savelkoul

**Affiliations:** ^1^Cell Biology and Immunology Group, Wageningen University, Wageningen, Netherlands; ^2^Top Institute Food and Nutrition, Wageningen, Netherlands; ^3^Gut Health and Food Safety, Institute of Food Research, Norwich, UK; ^4^Host Microbe Interactomics, Wageningen University, Wageningen, Netherlands; ^5^Laboratory of Microbiology, Wageningen University, Wageningen, Netherlands; ^6^Human Nutrition, Wageningen University, Wageningen, Netherlands; ^7^Department of Molecular Genetics, Erasmus University Medical Center, Rotterdam, Netherlands; ^8^CECAD Forschungszentrum, Universität zu Köln, Köln, Germany; ^9^University of Groningen, Groningen, Netherlands; ^10^Department of Immunology, Erasmus University Medical Center, Rotterdam, Netherlands; ^11^Department of Experimental and Clinical Medicine, University of Florence, Florence, Italy; ^12^Department of Pulmonary Medicine, Erasmus University Medical Center, Rotterdam, Netherlands

**Keywords:** aging, probiotics, immunity, mucus, intestinal barrier, microbiota

## Abstract

Although it is clear that probiotics improve intestinal barrier function, little is known about the effects of probiotics on the aging intestine. We investigated effects of 10-week bacterial supplementation of *Lactobacillus plantarum* WCFS1, *Lactobacillus casei* BL23, or *Bifidobacterium breve* DSM20213 on gut barrier and immunity in 16-week-old accelerated aging *Ercc1^−/Δ7^* mice, which have a median lifespan of ~20 weeks, and their wild-type littermates. The colonic barrier in *Ercc1^−/Δ7^* mice was characterized by a thin (< 10 μm) mucus layer. *L. plantarum* prevented this decline in mucus integrity in *Ercc1^−/Δ7^* mice, whereas *B. breve* exacerbated it. Bacterial supplementations affected the expression of immune-related genes, including Toll-like receptor 4. Regulatory T cell frequencies were increased in the mesenteric lymph nodes of *L. plantarum*- and *L. casei*-treated *Ercc1^−/Δ7^* mice. *L. plantarum*- and *L. casei*-treated *Ercc1^−/Δ7^* mice showed increased specific antibody production in a T cell-dependent immune response *in vivo*. By contrast, the effects of bacterial supplementation on wild-type control mice were negligible. Thus, supplementation with *L. plantarum* – but not with *L. casei* and *B. breve –* prevented the decline in the mucus barrier in *Ercc1^−/Δ7^* mice. Our data indicate that age is an important factor influencing beneficial or detrimental effects of candidate probiotics. These findings also highlight the need for caution in translating beneficial effects of probiotics observed in young animals or humans to the elderly.

## Introduction

Aging is accompanied by multiple age-related diseases (1), posing a major burden to public health care (2). With age, a decline in the regenerative potential of tissues due to stem cell exhaustion occurs (3). Turnover in epithelial cells is rapid, and mounting evidence indicates that intestinal stem cells are compromised with aging (4). For example, a crucial component of the intestinal barrier is mucus secreted by goblet cells (5). The Muc2 glycoprotein regulates immunity by inducing tolerogenic signals in mucosal dendritic cells (6) and is important in host–microbe interactions (7). Thus, changes in mucus quantity and integrity influence immunity (6, 8).

Aging is accompanied by the development of a low-grade inflammation (“inflammaging”), which is characterized by elevated IL-6 and TNF serum levels in elderly (9). Involution of the thymus and the bone marrow (BM) leads to decreased T and B cell production (10, 11). By contrast, the production of myeloid cells is enhanced with aging, characterized by a progressive increase of neutrophil frequencies in the circulation (12).

Probiotics are defined as live bacteria that confer health benefits to the host, for example, by competing with pathogens, enhancing intestinal barrier function, and regulating immunity (13, 14). They might, therefore, prevent some of the undesired age-related intestinal barrier and immune effects. Probiotic supplementation of elderly subjects led to changes in fecal microbiota composition (15–17), and affected the distribution and function of NK cells, macrophages, granulocytes, and T cells in the circulation (18, 19). Supplementation of aged mice with *Lactobacillus paracasei* resulted in increased IgG2a serum titers after antigenic challenge (20). Middle-aged mice that were supplemented with *Bifidobacterium animalis* showed decreased colon permeability, extended lifespan, and improved quality of life (21). Besides these studies, little is known about how exposure to probiotics impacts on the aging intestinal barrier and immune system. Moreover, it is unknown whether the beneficial effects of probiotics are age dependent.

In this report, we have used an accelerated aging mouse model to evaluate the effects of candidate probiotics in aging. Based on a variety of histological, functional, metabolomic, and proteomic data, it has been concluded that *Ercc1^−/Δ7^* mice resemble normal murine aging (22). Recently, we have shown that the immune system of *Ercc1^−/Δ7^* mice resembles the immune system of aged WT mice. For instance, we showed a similar decrease in B cell precursors and naïve T cells, and a similar increase in memory T cells and regulatory T cells (23). The ERCC1 protein is involved in multiple DNA repair pathways. *Ercc1^−/Δ7^* mice (median lifespan ~20 weeks) are deficient for fully functional ERCC1 protein. The expression of ERCC1-XPF (excision repair cross-complementation group 1-xeroderma pigmentosum group F) DNA repair endonuclease is reduced to ~5% compared with *Ercc1^+/+^* mice. Moreover, the residual ERCC1-XPF protein present is expressed from a truncated allele, and lacks the last seven amino acids. A reduction of ERCC1 protein activity leads to increased accumulation of DNA damage and, hence, results in an accelerated aging phenotype (24, 25).

The aim of this study was to investigate the potential of supplementation with candidate probiotic strains to ameliorate the effects of aging on the intestinal barrier and the immune system. Previously, probiotic activity was documented for *Lactobacillus plantarum* WCFS1 (26–28), *Lactobacillus casei* BL23 (29, 30), and relatives of *Bifidobacterium breve* DSM20213 (31). We selected these strains on the basis of induced IL-10/TNF ratios in young and aged immune cells *in vitro* (32). The three strains can be classified as potential pro-inflammatory (*L. plantarum*), regulatory (*L. casei*), or anti-inflammatory (*B. breve*), based on low, intermediate, or high IL-10/TNF ratios, respectively.

For this study, we supplemented 6-week-old *Ercc1^+/+^* mice and *Ercc1^−/Δ7^* mice with *L. plantarum, L. casei*, or *B. breve* for 10 weeks. Mucus barrier, microbiota composition, and gene regulation in the colon were analyzed, as well as the distribution of immune cells in various mucosal and peripheral lymphoid organs. We determined immune competence by antigenic challenge.

## Materials and Methods

### Mice

**Figure 1:**
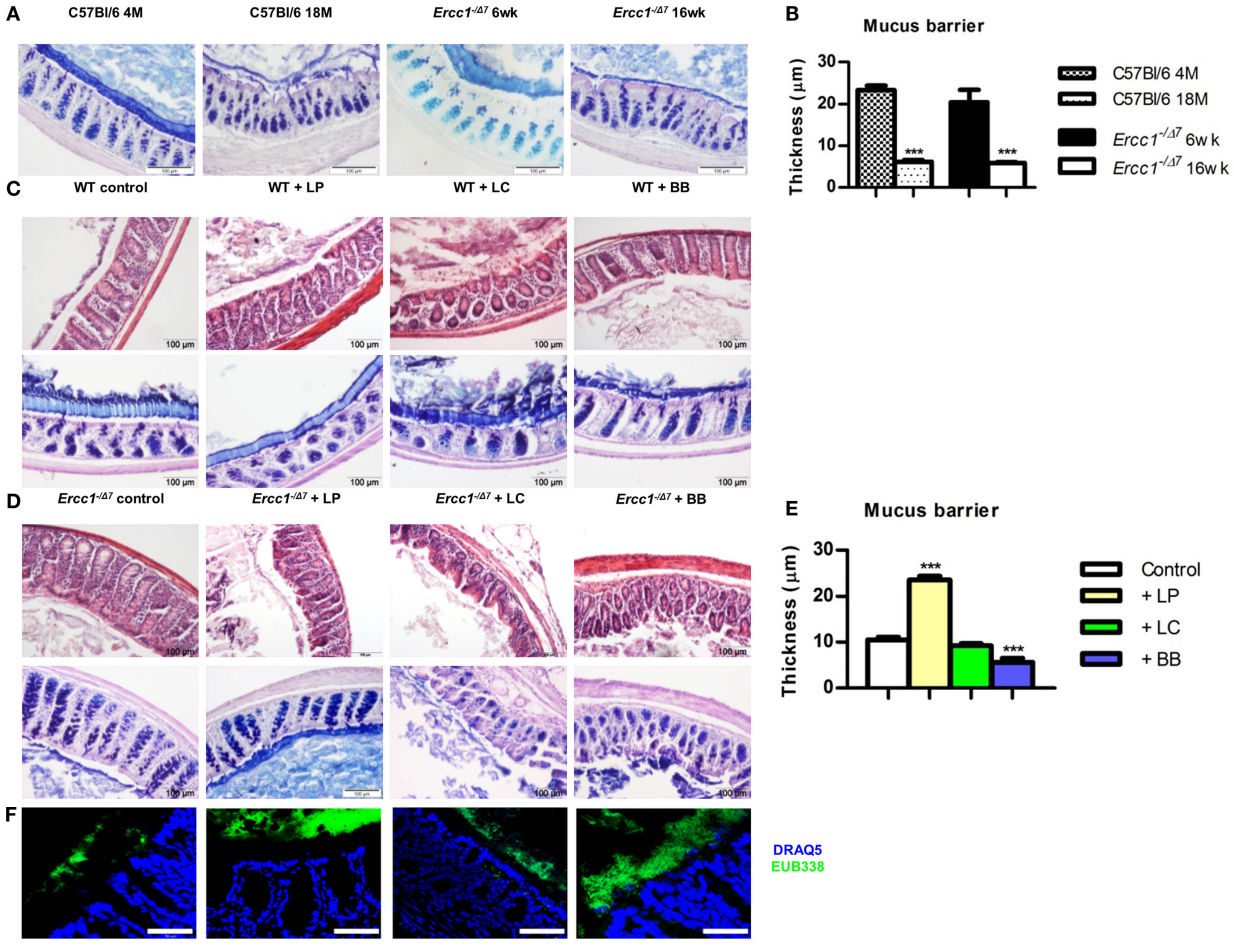
**Treatment with *L. plantarum* prevented the age-related decline in colonic mucus barrier of accelerated aging *Ercc1^−/Δ7^* mice**. **(A)** Representative pictures of proximal colon stained with PAS/Alcian Blue of 4-month-old (4 M) or 18-month-old (18 M) WT mice and 6-week-old (6 weeks) or 16-week-old (16 weeks) *Ercc1^−/Δ7^* mice. **(B)** Quantitative measurement of mucus thickness in young and old WT and *Ercc1^−/Δ^* mice by ImageJ. **(C)** Representative pictures of proximal colon stained with H&E or PAS/Alcian Blue of *Ercc1^+/+^* mice supplemented with control treatment, *L. plantarum* WCFS1 (LP), *L. casei* BL23 (LC), or *B. breve* DSM20213 (BB). **(D)** Representative pictures of proximal colon stained with H&E and PAS/Alcian Blue of *Ercc1^−/Δ7^* mice supplemented with control treatment, LP, LC, or BB. **(E)** Quantitative measurement of mucus thickness in *Ercc1^−/Δ7^* mice by ImageJ. **(F)** Fluorescence *in situ* hybridization (FISH) of proximal colon from *Ercc1^−/Δ7^* mice. Data represent the mean + SEM from four to six animals per group. ***p < 0.001. Scale bars histological pictures: 100 μm; scale bars FISH: 50 μm.

The generation and characterization of *Ercc1^+/Δ7^* and *Ercc1^−/+^* mice has been previously described (25). *Ercc1^−/Δ7^* mice were obtained by crossing *Ercc1^+/Δ7^* with *Ercc1^−/+^* mice of pure C57Bl6/J and FVB backgrounds to yield *Ercc1^−/Δ7^* with an F1 C57Bl6J/FVB hybrid background. Genotyping was performed as described previously (33). Wild-type littermates (C57Bl6J/FVB F1) were used as controls. Four-month-old and 18-month-old C57Bl6/J mice were purchased from Harlan (Horst, The Netherlands; only used in Figure 1).

Animals were housed in individual ventilated cages under SPF conditions. Experiments were performed in accordance with the Principles of Laboratory Animal Care and with Dutch legislation. This study was carried out in accordance with the recommendations of the Dutch Ethical Committee of Wageningen that approved the work. Blood was taken from mice being sacrificed, and serum was frozen in −80°C for later use. After mice (*n* = 4–6) were sacrificed, feces from colon was collected and snap-frozen. Distal ileum and proximal colon sections were isolated and fixed in Carnoy or snap-frozen in liquid nitrogen. BM, thymus, spleen, mesenteric lymph nodes (MLN), and Peyer’s patches (PPs) were isolated.

### Bacterial Cultures and Supplementation

*L. plantarum* WCFS1, *L. casei* BL23, and *B. breve* DSM20213 were grown on MRS medium (Merck, Darmstadt, Germany) until stationary phase, frozen in glycerol, and stored in −80°C until use. Upon use, bacteria were thawed and 10× diluted in NaHCO3/PBS buffer. Around 2 × 10^8^ CFU in 200 μL were administered to mice by gavage, three times per week. Treatment of mice started at 6 weeks of age until 1 day before sacrifice at 16 weeks or until death.

### Histology and Fluorescence *In Situ* Hybridization

Carnoy-fixed proximal colon sections were embedded in paraffin. Paraffin sections (5 μm) were attached to poly-l-lysine-coated glass slides (Thermo Scientific, Germany). After overnight incubation at 37°C, slides were de-waxed and rehydrated. Sections were stained with hematoxylin and eosin (H&E) and PAS/Alcian blue. Mucus layer thickness was measured using ImageJ software (NIH, MD, USA), as previously published (34). For detection of bacteria, tissue sections were used for fluorescence *in situ* hybridization (FISH), as previously published (8).

### MIT-Chips/16S Sequencing

Microbiota composition in colonic content was analyzed by Mouse Intestinal Tract Chip (MITChip), as described previously (35). The data were normalized and analyzed using a set of R-based scripts in combination with a custom-designed relational database, which operates under the MySQL database management system. For the microbial profiling, the Robust Probabilistic Averaging signal intensities of 2667 specific probes for the 94 genus-level bacterial groups detected on the MITChip were used (36). Diversity calculations were performed using a microbiome R-script package (https://github.com/microbiome). Multivariate statistics, redundancy analysis (RDA), and principal response curves were performed in Canoco 5.0 and visualized in triplots or a principal response curves plot (37).

### RNA Isolation and Transcriptome Analysis

Total RNA was isolated from proximal colon (*n* = 3–6 per group) using the RNeasy kit (Qiagen) with a DNase digestion step according to the manufacturer’s protocol. Transcriptome analysis on individual samples was performed as previously described (8). The gene expression datasets were deposited in NCBI’s Gene Expression Omnibus (GEO) and are accesible through GEO Series accession number: GSE87368.

### General Flow Cytometry Procedures

Single-cell suspensions of BM were obtained by crushing femurs, tibias, iliac crests, and sternum with mortar and pestle. BM cells were then filtered on a 40-μm cell strainer. A proportion of the BM cells was frozen for later use in *in vitro* cultures. Spleen, MLN, PP, thymus, and peritoneal cavity single-cell suspensions were obtained by gently pushing cells through a 40-μm cell strainer with a syringe. All cells were stained for extracellular markers and dead cells were identified with fixable live/dead stain (Ebioscience, San Diego, CA, USA), after which intracellular staining was enabled by fixing and permeabilizing cells with Fix/Perm buffer (Ebioscience) according to manufacturer’s instructions. Antibodies used for flow cytometric measurements are listed in Supplementary Table 1 in Data Sheet 1. All flow cytometric measurements were performed on a Canto II flow cytometer (BD Biosciences, Erembodegem, Belgium). FlowJo vX.07 software (Tree Star) was used for data analysis. Gating of all presented immune cell populations was based on single live cells.

### Spleen Cell Cultures

Splenic cells were cultured at 10^6^ cells/mL for 4 days in the absence or presence of 5 μg/mL concanavalin A (ConA). Proliferation was measured by Ki-67 (Ebioscience). Supernatants were stored at −20°C. After thawing, levels of IL-2, IL-4, IL-6, IL-10, IL-17A, IFN-γ, and TNF were measured with the Cytometric Bead Array Th1/Th2/Th17 Kit (BD Biosciences), according to manufacturer’s instructions. Samples were acquired on a Canto II flow cytometer. Data were analyzed using FCAP Array version 3.0 (BD Biosciences) software.

### Antibody Titers in Serum

Levels of IgM, IgG1, IgG2a, IgG2b, IgG3, IgE, and IgA were analyzed in serum using ProcartaPlex Mouse Antibody Isotyping Panel kit on the Luminex platform (Affymetrix, Santa Clara, CA, USA) according to the manufacturer’s instructions. Data were acquired on a BioPlex 200 (Bio-Rad, Hercules, CA, USA) and analyzed with BioPlex software (version 5.0, Bio-Rad).

### *In Vivo* Immunization and Antibody Detection

Primary and secondary T cell-dependent (TD) immune responses against TNP-KLH were measured 7 days after primary i.p. immunization and 7 days after i.p. booster immunization. The primary immunization was performed at 8 weeks of age (TNP-KLH in alum), booster doses were injected at 12 weeks of age (TNP-KLH in PBS). Total and TNP-specific Ig subclasses were determined by sandwich ELISA as previously described (38).

### Statistical Analysis

Values are expressed as mean + SEM. Normal distribution of the data was confirmed using the Kolmogorov–Smirnov test. Statistical comparisons were performed using the two-sided Student’s *t*-test. Where non-Gaussian distribution was demonstrated, we applied the non-parametric Mann–Whitney U test. Where no equal variances were observed, we applied the two-sided Student’s *t*-test with Welch’s correction. Statistical comparisons for lifespan data were performed using the log-rank (Mantel–Cox) test. Statistical comparisons for serum immunoglobulins were performed using two-way ANOVA, with subsequent Bonferroni posttests. Values of *p* < 0.05 were considered to be statistically significant. Values between *p* > 0.05 and *p* < 0.10 were considered as a trend.

## Results

### The Mucus Layer in the Colon Declines with Age

To assess the mucus barrier in normal and accelerated aging, we compared the proximal colon of 4-month-old (young) with 18-month-old (aged) C57Bl/6 mice, and of 6-week-old (young) with 16-week-old (aged) *Ercc1^−/Δ7^* mice. We observed that in aged C57Bl/6 and *Ercc1^−/Δ7^* mice, a thinner mucus layer was present, compared with young C57Bl/6 and *Ercc1^−/Δ7^* mice (Figure 1A). With ImageJ, we measured the thickness of the mucus layer. In young C57Bl/6 and *Ercc1^−/Δ7^* mice, a mucus layer of ~20 μm was present, whereas in normal and accelerated aged mice, a significantly thinner mucus layer of less than 10 μm was observed (*p* < 0.001; Figure 1B).

### Bacterial Supplementations Do Not Change the Mucus Layer in Colon of Young WT Mice

To determine the effects of the three selected bacterial strains in the young intestine, we analyzed proximal colon tissues of WT mice that were treated with *L. plantarum* WCFS1, *L. casei* BL23, or *B. breve* DSM20213 for 10 weeks. No change in tissue integrity (H&E) or mucus layer (PAS/Alcian Blue) was observed in the colon after supplementation with bacterial strains (Figure 1C).

### Age-Related Decline in the Mucus Barrier is Prevented by Supplementation of *Ercc1^−/Δ7^* Mice with *L. plantarum*

Because the mucus layer declines with age, we questioned whether bacterial supplementation of *Ercc1^−/Δ7^* mice prevents the decline in mucus barrier. Colon tissue of 10-week treated *Ercc1^−/Δ7^* mice was checked for tissue integrity and mucus layer thickness. In contrast to our findings in WT mice, bacterial supplementation had significant effects on tissue integrity and the mucus layer. In *Ercc1^−/Δ7^* mice supplemented with *L. plantarum*, the colon showed a thicker mucus layer than their controls (Figure 1D). *L. plantarum* supplementation completely prevented age-related decline in the mucus layer compared with controls (*p* < 0.001; Figure 1E), resulting in a mucus thickness comparable to young WT mice. Spatial compartmentalization of bacteria in the colon was improved after *L. plantarum* supplementation (Figure 1F), as demonstrated by FISH analyses. On the contrary, *Ercc1^−/Δ7^* mice supplemented with *L. casei* or *B. breve* showed loss of tissue integrity (Figure 1D). No difference in mucus thickness was observed after supplementation with *L. casei* (Figure 1E). *B. breve* supplementation resulted in a deteriorated mucus layer and a loss in mucus thickness (*p* < 0.001; Figure 1E). *B. breve* supplementation also resulted in less spatial compartmentalization of bacteria in the colon of *Ercc1^−/Δ7^* mice (Figure 1F).

Collectively, these data show that *L. plantarum* supplementation improves the mucus layer in the aged (but not young) colon. In addition, supplementation with *L. casei* or *B. breve* exacerbates the age-related decline of mucus barrier in the colon.

### Bacterial Supplementation Associated with Minor Alterations in Colonic Microbiota Composition

As we introduced bacteria by bacterial supplementations into the intestinal microbial community, we investigate whether changes in the microbiota composition were underlying the observed changes in the mucus barrier of *Ercc1^−/Δ7^* mice. Microbiota composition was determined by performing 16S rRNA gene microbiota profiles of colonic content. The bacterial supplementations did not significantly alter microbial diversity nor richness (data not shown).

**Figure 2:**
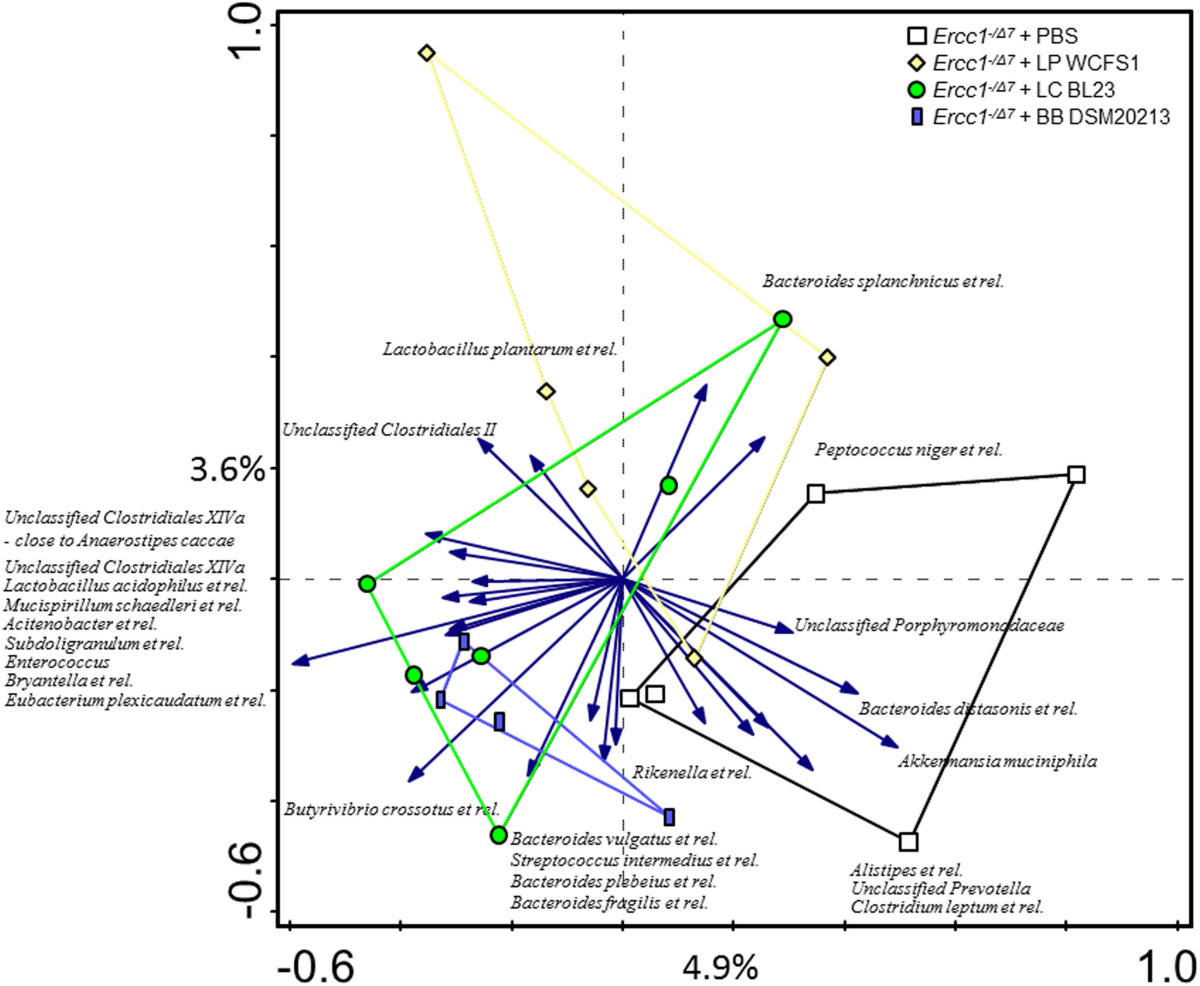
**The effect of bacterial supplementations on colonic microbiota composition in *Ercc1^−/Δ7^* mice**. Redundancy analysis of the microbial composition after bacterial supplementations, on genus-like level of the MITChip analysis. Mice belonging to control-, LP-, LC-, and BB-treated groups are indicated by white squares, yellow diamonds, green circles, and blue rectangles, respectively. First and second ordination axes are plotted, showing 4.9 and 3.6% of the variability in the dataset, respectively. No significant changes were observed. LP, *L. plantarum* WCFS1; LC, *L. casei* BL23; BB, *B. breve* DSM20213.

Redundancy analysis showed that 10.1% of the total variability of the gut microbiota can be related to the bacterial supplementations (Figure 2). No statistical significance was established. The first ordination axis explained 4.9% of the variability and separated *Ercc1^−/Δ7^* mice supplemented with either of the three bacterial strains from the control *Ercc1^−/Δ7^* mice. The second ordination axis explained 3.6% of the variability but did not result in a separation between groups. The third ordination axis explained an additional 1.6% of the variability (data not shown).

**Figure 3:**
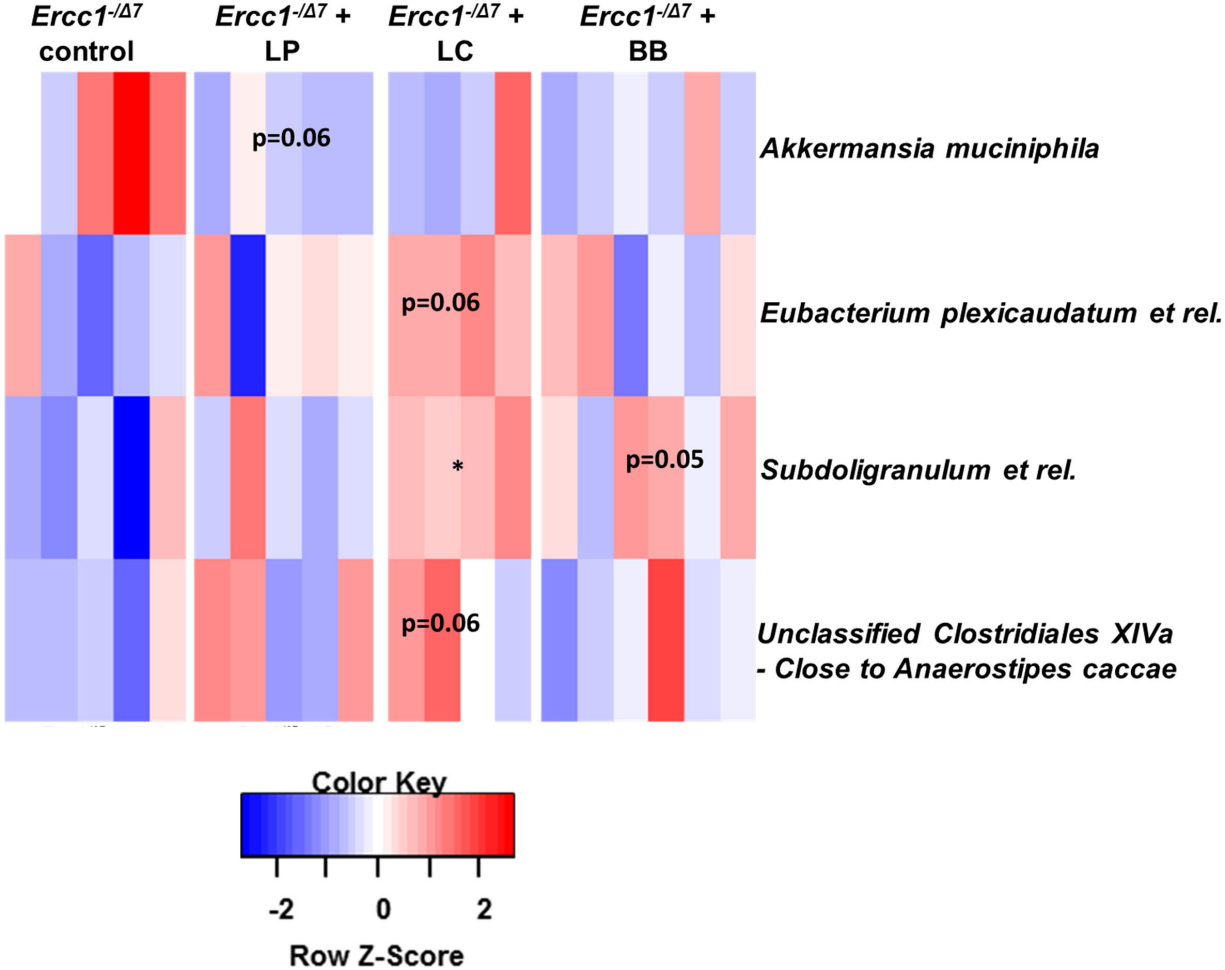
**Bacterial supplementation induced changes in bacterial taxa in the colon of *Ercc1^−/Δ7^* mice**. Wilcoxon tests comparing mice treated with bacterial strains with control group. Data represent *n* = 4–6 mice per group. *p < 0.05.

To assess whether significant changes in the microbial genus-like bacterial groups existed after different bacterial supplementations in *Ercc1^−/Δ7^* mice, we performed the Wilcoxon test. *Subdoligranulum* was higher in mice supplemented with *L. casei* (*p* < 0.05), whereas it tended to be higher in mice supplemented with *B. breve* (*p* = 0.05), as compared with control mice (Figure 3). *Akkermansia muciniphila* tended to be less present (*p* = 0.06) in mice supplemented with *L. plantarum* compared with control mice. *Eubacterium plexicaudatum* and a close relative to *Anaerostipes caccae* tended to be higher (*p* = 0.06) in *Ercc1^−/Δ7^* mice supplemented with *L. casei*.

These data demonstrate some differences in microbial species between control-treated *Ercc1^−/Δ7^* mice and *Ercc1^−/Δ7^* mice treated with bacterial supplementations.

### Distinct Gene Expression Profiles in Colon after Each Bacterial Supplementation

**Figure 4:**
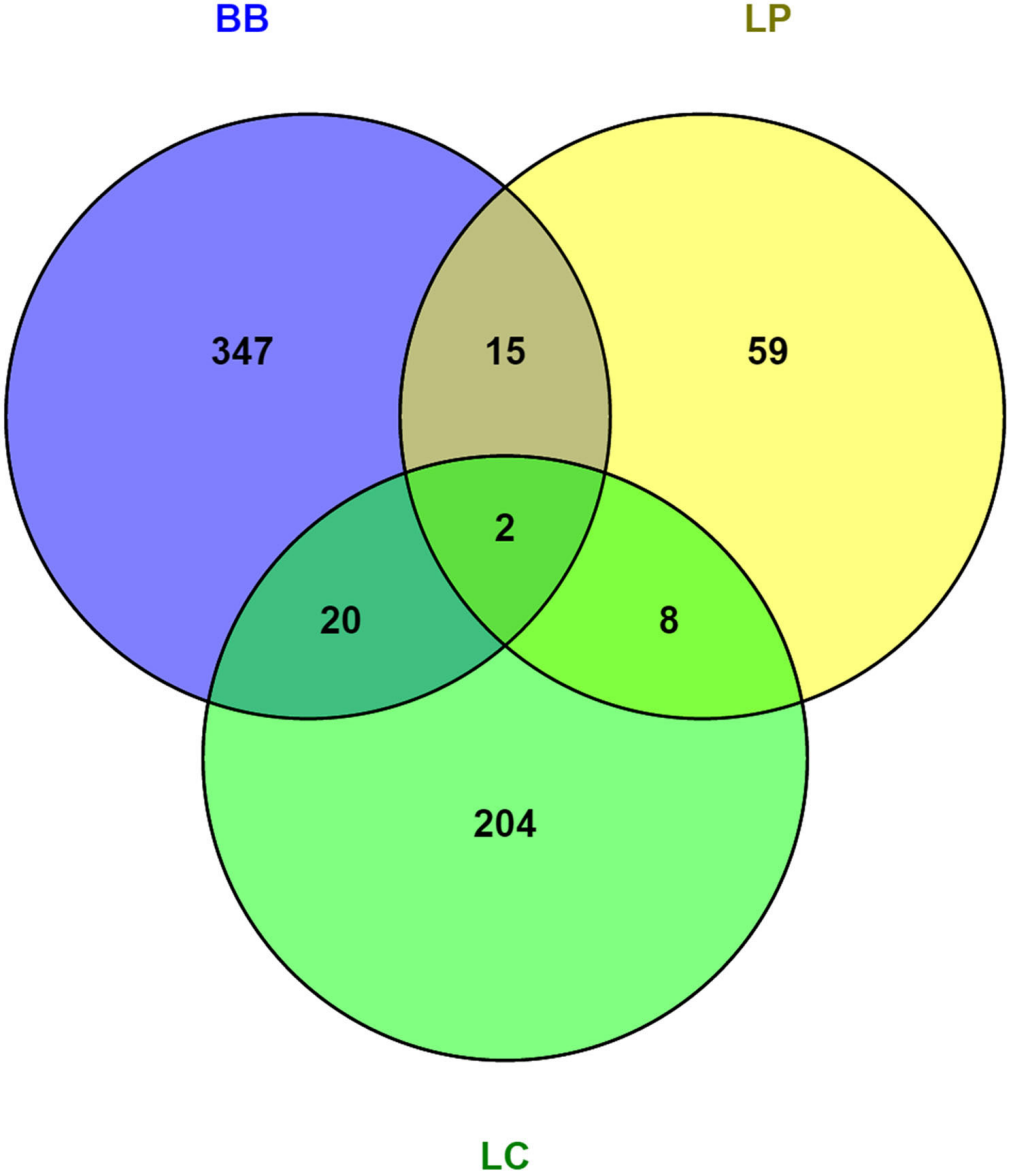
**Venn diagrams of differentially regulated genes in colon of *Ercc1^−/Δ7^* mice after bacterial supplementations**. Total number of genes altered in the proximal colon of *Ercc1^−/Δ7^* mice treated with *L. plantarum* WCFS1 (LP), *L. casei* BL23 (LC), or *B. breve* DSM20213 (BB) compared with control-treated *Ercc1^−/Δ7^* mice. Venn diagram of the total number of genes upregulated and downregulated in the proximal colon of *Ercc1^−/Δ7^* mice treated with LP, BB, or LC (*p* < 0.05 and >1.2-fold difference).

To understand the mechanisms by which bacterial supplementation changes the mucus barrier, we performed transcriptome analysis on the proximal colon of *Ercc1^−/Δ7^* mice. Gene expression microarrays on total proximal colon samples from *Ercc1^−/Δ7^* mice treated with bacterial supplementations or control treatment revealed relatively low numbers of differentially expressed genes: 84 by *L. plantarum*, 238 by *L. casei*, and 384 by *B. breve*. Only a few genes were overlapping between two or three different bacterial supplementations, whereas most of the differentially expressed genes were distinctly regulated by one of the treatments (Figure 4).

Several growth- and immune-related genes were differentially expressed after bacterial supplementation. Apolipoprotein (APO) A-1, APOA-4, suppressor of cytokine signaling (SOCS) 3, and toll-like receptor (TLR) 4 were upregulated more than 1.2-fold after *L. plantarum* supplementation (Data Sheet 2 in the Supplementary Material). Several immunoglobulin variable genes and TLR13 were upregulated after administration of *L. casei*, whereas defensin 40β was 1.3-fold downregulated. Defensin 24α, amphiregulin, and keratinocyte growth factor 7 (FGF7) were upregulated more than 1.4-fold after administration of *B. breve*, while TLR6, TLR7, and CCL3 (MIP-1α) were more than 1.2-fold downregulated (Data Sheet 2 in the Supplementary Material). Remarkably, we found no significant up- or downregulation in any mucin.

### Bacterial Supplementation Alters Growth- and Immune-Related Pathways in Colon

Because we found relatively low numbers of differentially expressed genes, we applied a gene set enrichment analysis (GSEA) (39) to gain insight into the regulated pathways by bacterial supplementations. Upstream regulators that can explain the observed changes in gene expression were identified using Ingenuity Upstream Regulator Analysis.

Gene set enrichment analysis revealed that *L. plantarum* supplementation significantly enhanced several processes involved in growth and cell cycle, and immunity (Supplementary Table 2 in Data Sheet 1), such as “Type II Interferon Signaling,” “VIP pathway,” and “IL8/CXCR1 pathway.” Interestingly, in the top-10 of upregulated pathways, three pathways were linked to DNA repair: the “Fanconi Pathway,” “ATRBRCA pathway,” and the “Fanconi anemia pathway.” Several growth factors were activated after *L. plantarum* supplementation: leptin, epidermal growth factor (EGF), platelet-derived growth factor (PDGF) BB, early growth response protein (EGR) 1, and insulin-like growth factor (IGF) 1 (Table 1). Inflammatory cytokines (IFN-γ, IL-1β, IL-4, TNF) and CD40L (CD154) were activated in colon of mice supplemented with *L. plantarum*, compared with colon of mice supplemented with control treatment.

**Table 1:** **Activation *z*-scores of upstream regulators in proximal colon of *Ercc1^−/Δ7^* mice after bacterial supplementations *L. plantarum* WCFS1 (LP), *L. casei* BL23 (LC), or *B. breve* DSM20213 (BB) as determined by Ingenuity**.

Upstream regulator	LP	LC	BB
Leptin	2.41		
EGF	2.36		3.36
IL4	2.18		
IFN-γ	2.00		−1.35
PDGF BB	2.00		1.15
P38 MAPK	1.97		
CD40L	1.96		
Palmitic acid	1.96		
EGR1	1.95		
IGF1	1.82		
IL1β	1.77		
Ethanol	1.76		
CREB1	1.55		
CREBBP	1.54		
TNF	1.53		
KLF4		2.04	
Resistin-like β		2.00	
PML		−1.73	
miR-4800-5p		−1.98	
GATA3		−1.98	
MTOR		−2.00	
miR-4455		−2.22	
ADCYAP1			2.60
EDN1			2.17
WNT3A			2.16
VIP			1.95
FGF2			1.74
GLI1			1.63
miR-6967-5p			−1.58
Klra7 (includes others)			−1.87
IgG			−1.89
EZH2			−1.96
GATA2			−2.00
ANXA7			−2.00
miR-4707-5p			−2.16
ITK			−2.19
miR-4459			−2.63

*Upstream regulators involved in growth and cell cycle are highlighted in blue; upstream regulators involved in immunity are highlighted in orange. Cut-off values for activation z-score ≥1.5 or ≤−1.5 combined with p < 0.05. Activated in blue, inhibited in red*.

*ADCYAP, adenylate cyclase activating polypeptide; ANX, annexin; CREB(BP), cAMP-responsive element (binding protein); EDN, endothelin; EGF, epidermal growth factor; EGR, early growth response protein; EHZ, enhancer of zeste homolog; FGF, fibroblast growth factor; GLI, glioma-associated oncogene family zinc finger; IFN, interferon; IGF, insulin-like growth factor; ITK, IL-2-inducible T cell kinase; KLF, Kruppel-like factor; Klra, killer cell lectin-like receptor, subfamily A; LEP, leptin; MTOR, mechanistic target of rapamycin; PDGF, platelet-derived growth factor; PML, promyelocytic leukemia protein; RETNLB, resistin-like β; TNF, tumor necrosis factor; VIP, vasoactive intestinal peptide; WNT, wingless-type MMTV integration site family*.

*Lactobacillus casei* supplementation enhanced several processes involved in growth and cell cycle, like “Mitotic G1-G1 S Phases,” “DNA replication,” “Synthesis of DNA,” and “G1 S transition” (Supplementary Table 2 in Data Sheet 1). In addition, the “NOD-like receptor signaling pathway” was enhanced after *L. casei* supplementation, as well as and the “Unfolded protein response” (UPR), indicated endoplasmatic reticulum (ER) stress. Upstream regulators resistin-like β (RTNLB; activated) and GATA3 (inhibited) were regulated in the colon of mice supplemented with *L. casei* (Table 1).

Several metabolic pathways were enhanced in colon of *B. breve*-supplemented mice (Supplementary Table 2 in Data Sheet 1). Of note, “Protein folding” was upregulated. In contrast to *L. plantarum* and *L. casei* supplementation, *B. breve* supplementation significantly inhibited several processes involved in immunity, such as “IL2 STAT5 pathway,” “Immunoregulatory interactions between lymphoid/non-lymphoid cells,” “Type II Interferon signaling,” “IL4 2 pathway,” and “IL6 7 pathway” (Supplementary Table 3 in Data Sheet 1). *B. breve* supplementation activated EGF and inhibited fibroblast growth factor (FGF) 2. IgG, GATA2, and IL-2-inducible T cell kinase (ITK) were inhibited in colon of mice supplemented with *B. breve*. In line with GSEA, IFN-γ was inhibited as well after *B. breve* supplementation.

These data indicate that immune pathways in the colon are enhanced by *L. plantarum* and *L. casei*, but are inhibited by *B. breve* supplementation.

### *L. plantarum* and *L. casei* Supplementation Induce Regulatory T Cells in MLN

Based on the regulation of immune genes by bacterial supplementations, we tested whether the distribution of immune cells was altered in mucosal immune organs of *Ercc1^−/Δ7^* mice.

**Figure 5:**
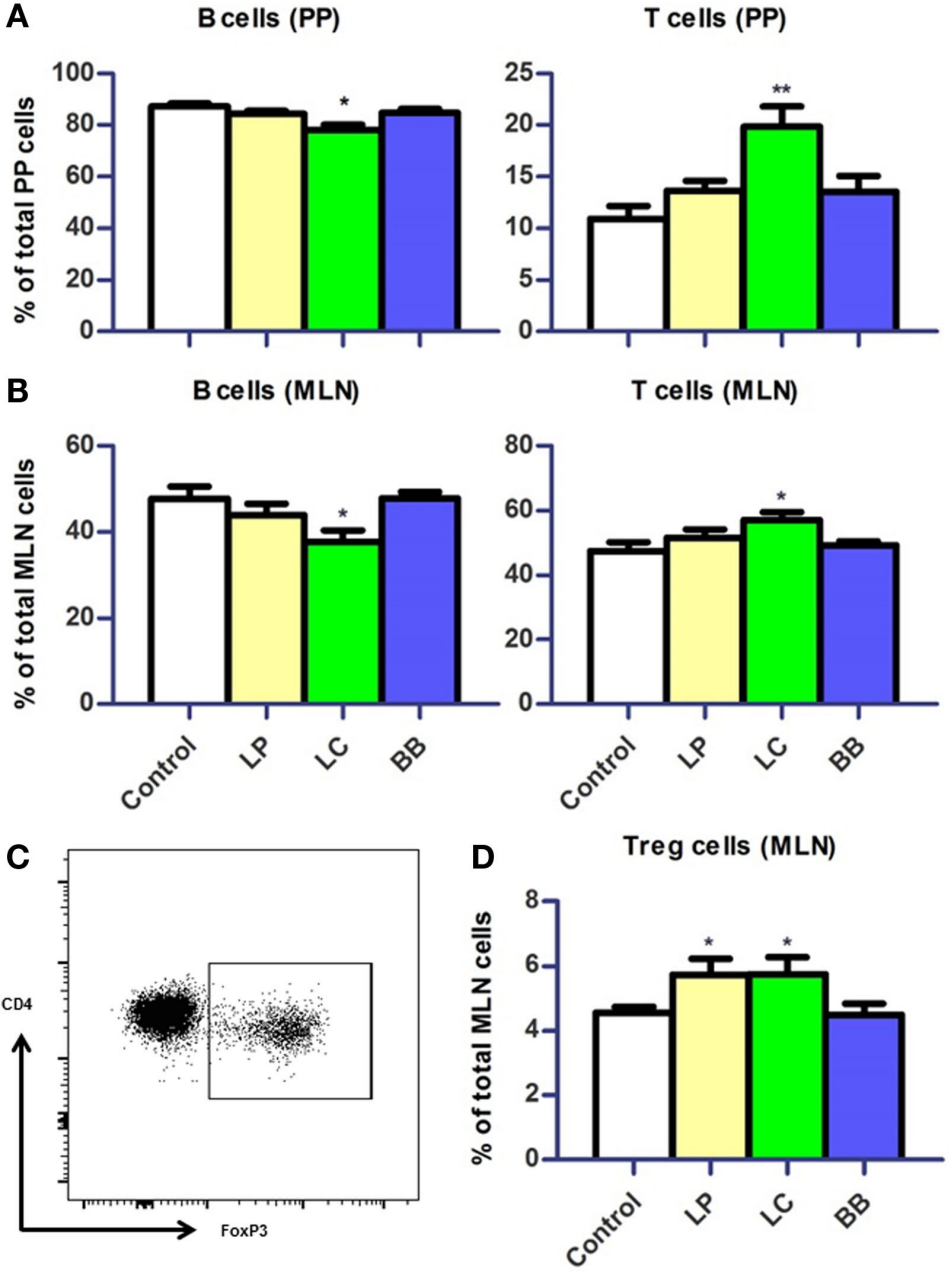
**Distribution of B cells and T cells in Peyer’s patches (PP) and mesenteric lymph nodes (MLN) upon bacterial supplementation in *Ercc1^−/Δ7^* mice**. **(A,B)** Mean frequencies of B and T cells in PP and MLN were determined by flow cytometry. B cells were defined as CD19^+^, T cells were defined as CD3^+^. **(C)** Flow cytometric analysis of CD3^+^CD4^+^CD8^−^ regulatory T (Treg) cells in MLN. **(D)** Mean frequencies of Treg cells in MLN. Data represent the mean + SEM from four to six animals per group. LP, *L. plantarum* WCFS1; LC, *L. casei* BL23; BB, *B. breve* DSM20213. *p < 0.05; **p < 0.01.

First, we evaluated changes in distribution of immune cells in PPs and MLN. B cell frequencies were reduced in PP and MLN (*p* < 0.05) after *L. casei* supplementation in *Ercc1^−/Δ7^* mice (Figure 5A). By contrast, frequencies of T cells were increased in PP (*p* < 0.01) and MLN (*p* < 0.05; Figure 5B). The frequencies of regulatory T (Treg) cells in MLN were increased after *L. plantarum* and *L. casei* supplementation (*p* < 0.05; Figures 5C,D). No changes in distribution of B and T cells were observed upon bacterial supplementation in WT mice, except for a tendency to decreased Treg cells after *L. casei* supplementation (*p* = 0.09; Supplementary Figure 1 in Data Sheet 1).

### *L. casei* Elevates Systemic Inflammatory Markers

**Figure 6:**
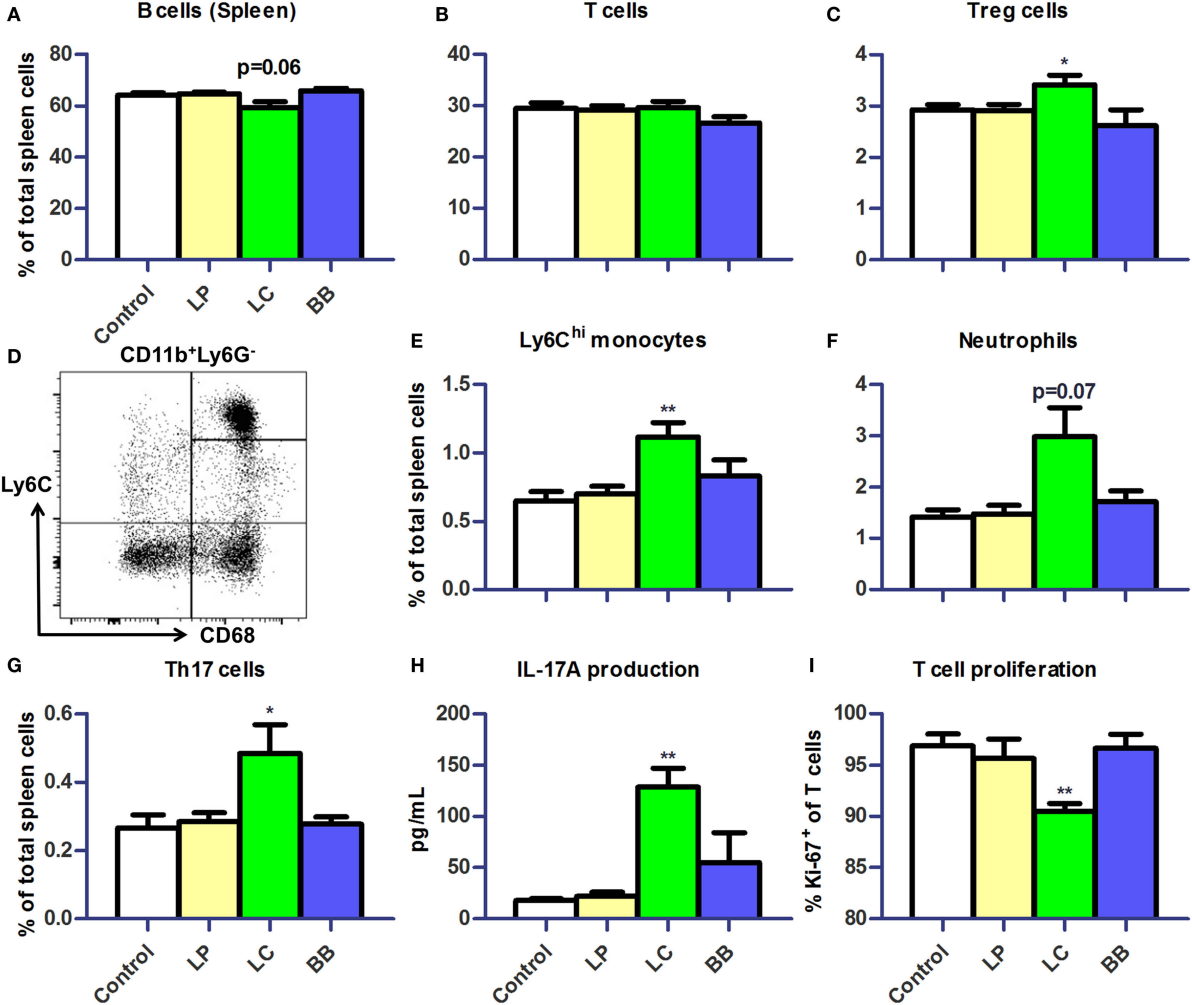
***L. casei* supplementation of *Ercc1^−/Δ7^* mice raised inflammatory markers in spleen**. **(A,B)** Mean frequencies of B and T cells in spleen were determined by flow cytometry. B cells were defined as CD19^+^, T cells were defined as CD3^+^. **(C)** Mean frequencies of Treg cells in spleen. **(D)** Flow cytometric analysis of splenic monocytes. CD11b^+^Ly6G^−^CD68^+^ cells were divided in Ly6C^hi^, Ly6C^int^, and Ly6C^lo^ monocytes. **(E–G)** Mean frequencies of Ly6C^hi^ monocytes, neutrophils, and CD3^+^CD4^+^CD8^−^Rorγt^+^ Th17 cells were determined by flow cytometry. **(H)** Mean concentration of IL-17A production by splenocytes stimulated with ConA for 4 days, as determined by Cytometric Bead Array. **(I)** Mean proliferating T cells (Ki-67^+^) in splenocyte culture stimulated with ConA for 4 days, as determined by flow cytometry. Data represent the mean + SEM from four to six animals per group. *p < 0.05; **p < 0.01. LP, *L. plantarum* WCFS1; LC, *L. casei* BL23; BB, *B. breve* DSM20213.

Next, we assessed distribution of immune cells in the spleen. We noted that the relative spleen weight increased after *L. casei* supplementation in *Ercc1^−/Δ7^* mice (Supplementary Figure 2A in Data Sheet 1). Absolute numbers of spleen cells were not affected by bacterial supplementations (data not shown). Splenic B cell frequencies tended to be decreased after *L. casei* supplementation (*p* = 0.06; Figure 6A), but no changes in T cell frequencies were observed (Figure 6B). Treg cell frequencies in the spleen were increased after *L. casei* supplementation in *Ercc1^−/Δ7^* mice (*p* < 0.05; Figure 6C).

Increased frequencies of CD11b^+^Ly6G^−^CD68^+^Ly6C^hi^ monocytes (*p* < 0.01; Figures 6D,E) and a tendency to increased frequencies of CD11b^+^CD68^int^Ly6C^int^Ly6G^+^ neutrophils were observed after *L. casei* supplementation (*p* = 0.07; Figure 6F). In addition, the proportions of CD3^+^CD4^+^RORγt^+^ Th17 cells (Supplementary Figure 2B in Data Sheet 1) were increased after *L. casei* supplementation (*p* < 0.05; Figure 6G). A 4-day culture of splenocytes stimulated with concanavalin A (ConA), also showed increased IL-17A production (*p* < 0.01; Figure 6H) and a decreased T cell proliferation in splenocytes derived from *L. casei*-treated mice (*p* < 0.01; Figure 6I). None of these changes were observed in *Ercc1^−/Δ7^* mice treated with *L. plantarum* or *B. breve*, and in WT mice treated with each of the bacterial supplementations (Supplementary Figure 3 in Data Sheet 1). These data suggest that *L. casei*, in contrast to *L. plantarum* and *B. breve*, raises several inflammatory markers in *Ercc1^−/Δ7^* mice.

### Lymphocyte and Myeloid Development Affected after *L. plantarum* or *L. casei* Supplementation

**Figure 7:**
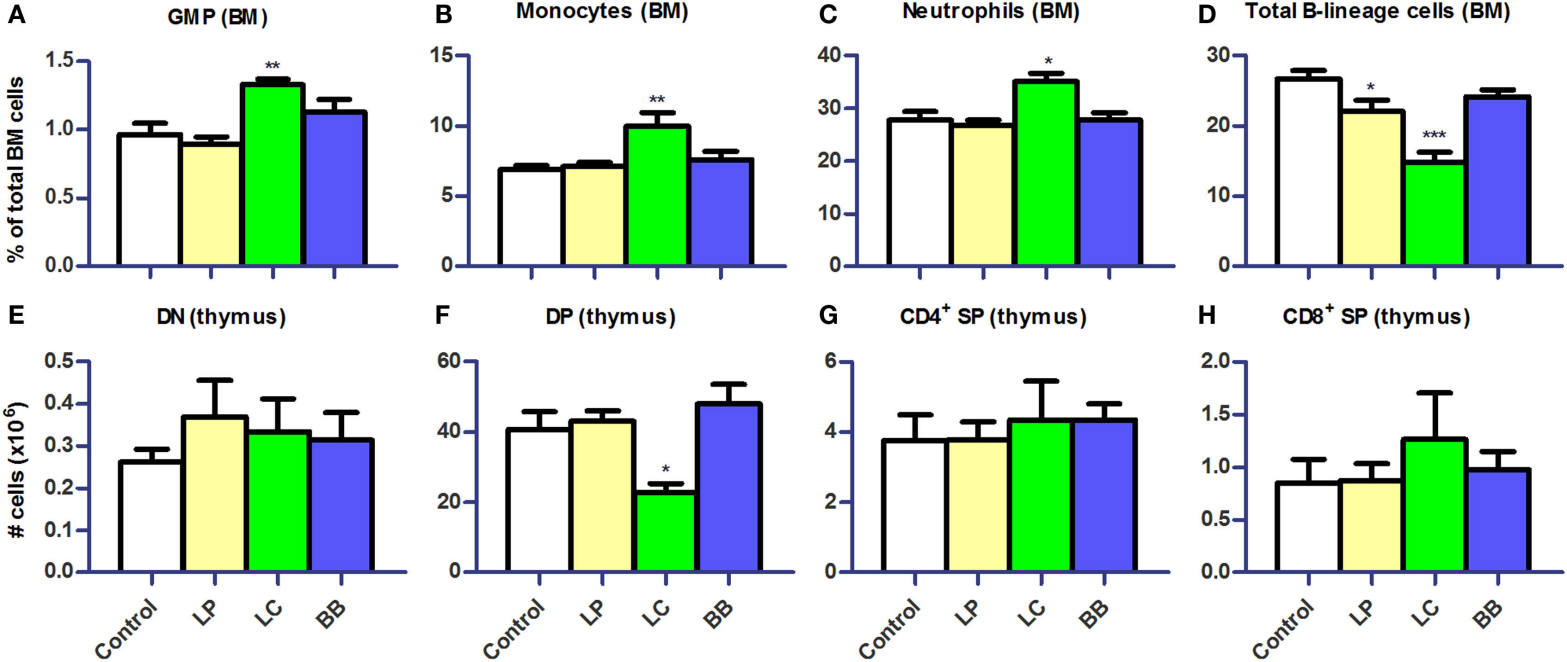
***L. casei* or *L. plantarum* supplementation altered myeloid and lymphoid development in bone marrow and thymus of *Ercc1^−/Δ7^* mice**. **(A–D)** Mean frequencies in bone marrow (BM) were determined by flow cytometry. Granulocyte–monocyte precursors (GMP) were defined as Lin^−^CD117^hi^CD11c^−^CD135^−^CD16/32^+^, neutrophils as CD11b^+^Ly6G^+^, monocytes as Ly6C^hi^CD31^−^, and B-lineage cells as CD19^+^CD45R^+^. **(E–H)** Mean absolute numbers were determined by cell counts and flow cytometry. Double-negative (DN) cells were defined as CD3^−^CD4^−^CD8^−^, double-positive (DP) cells as CD3^−^CD4^+^CD8^+^, CD4^+^ single-positive (SP) as CD3^+^CD4^+^CD8^−^, and CD8^+^ SP as CD3^+^CD4^−^CD8^+^. Data represent the mean + SEM from four to six animals per group. *p < 0.05; **p < 0.01; ***p < 0.001. LP, *L. plantarum* WCFS1; LC, *L. casei* BL23; BB, *B. breve* DSM20213.

We subsequently investigated the development of B cells and myeloid cells in BM and of T cells in thymus of *Ercc1^−/Δ7^* mice, as the observed changes in cell distribution in PP, MLN, and spleen might be explained by an altered migration or production. Absolute numbers in the BM were unchanged after bacterial supplementation (data not shown). In the BM, we observed significantly higher Lin^−^CD117^hi^CD11c^−^CD135^−^CD16/32^+^ granulocyte–monocyte precursor (GMP), CD11b^+^Ly6G^+^ neutrophil, and Ly6C^hi^CD31^−^ monocyte frequencies after *L. casei* supplementation (Figures 7A–C). Frequencies of total CD19^+^CD45R^+^ B-lineage cells were decreased after *L. plantarum* (*p* < 0.05) and *L. casei* supplementation (*p* < 0.001), but not after *B. breve* supplementation (Figure 7D). We observed a reduction in all B-lineage subsets, except pro-B cells, after *L. casei* and *L. plantarum* supplementation (data not shown). In thymus, only *L. casei* supplementation caused changes in cell distribution, with significantly reduced CD3^−^CD4^+^CD8^+^ double-positive (DP) cell numbers (Figures 7E–H).

In WT mice, we found no effect of bacterial supplementations on distribution of immune cells in BM or thymus, except for B-lineage cells after *L. plantarum* and *L. casei* supplementation (Supplementary Figure 4 in Data Sheet 1).

### Bacterial Supplementations Do Not Alter Lifespan of *Ercc1^−/Δ7^* Mice

The accelerated aging of *Ercc1^−/Δ7^* mice enabled us to assess the potential life-extending properties of the bacterial strains. No significant change in lifespan of *Ercc1^−/Δ7^* mice was observed after lifelong supplementation with *L. plantarum* or *L. casei* (Supplementary Figure 5 in Data Sheet 1).

### *L. casei* Supplementation Increases IgG Serum Titers

**Figure 8:**
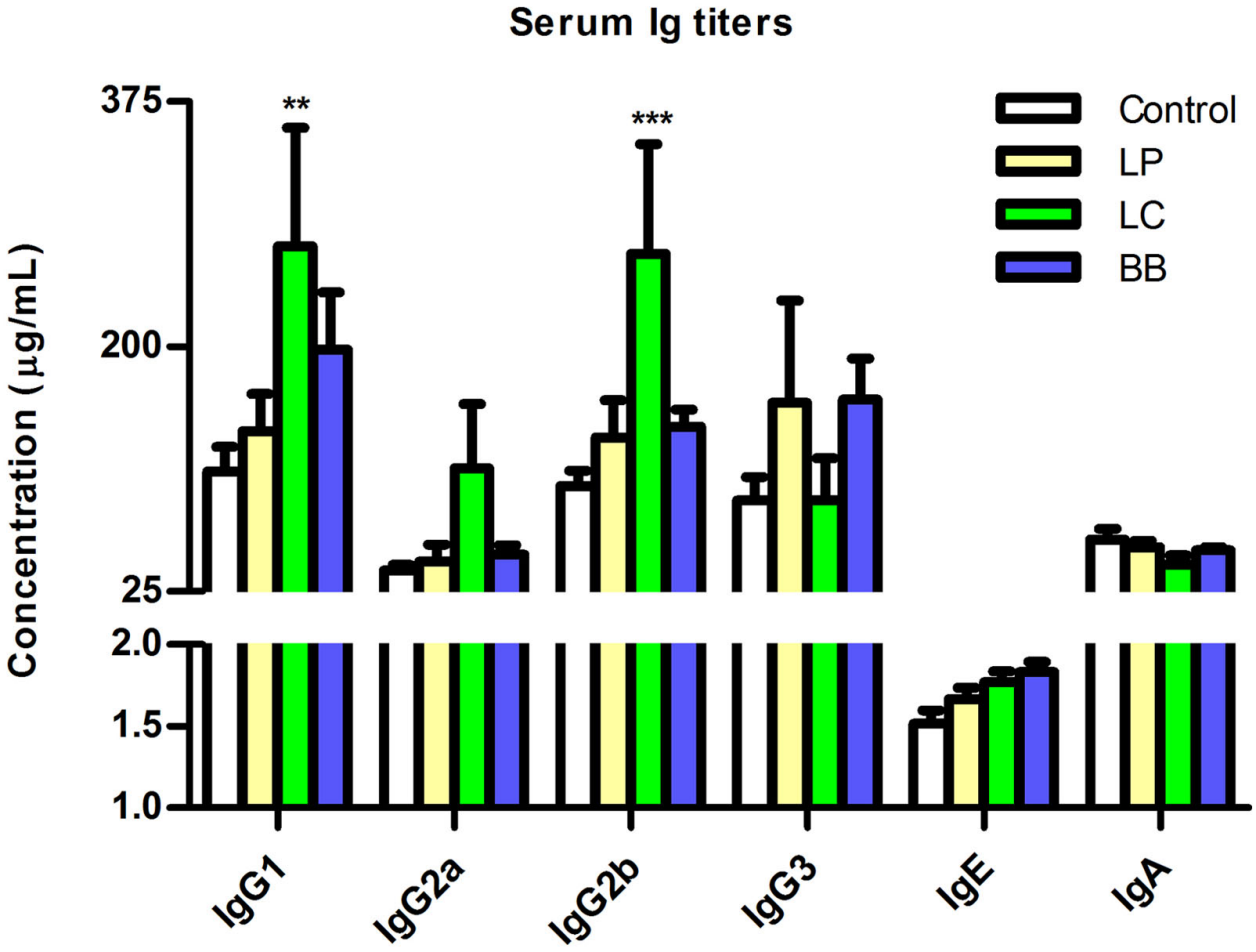
***L. casei* supplementation increased IgG1 and IgG2b titers in *Ercc1^−/Δ7^* mice**. Mean titers of IgG1, IgG2a, IgG2b, IgG3, IgE, and IgA in serum, as determined by Luminex. Data represent the mean + SEM from three to six animals per group. **p < 0.01; ***p < 0.001. LP = *L. plantarum* WCFS1; LC = *L. casei* BL23; BB = *B. breve* DSM20213.

Because *L. casei* supplementation led to decreased B cell proportions in several immune organs of *Ercc1^−/Δ7^* mice, we tested whether serum antibody titers in *Ercc1^−/Δ7^* mice were altered. Total IgG1 and IgG2b (but not IgG2a, IgG3, IgE, and IgA) titers were significantly increased after *L. casei* supplementation (Figure 8). *L. plantarum* and *B. breve* supplementation did not significantly change titers of any Ig subclass.

### Immune Competence Improved by *L. casei* and *L. plantarum* Supplementation

**Figure 9:**
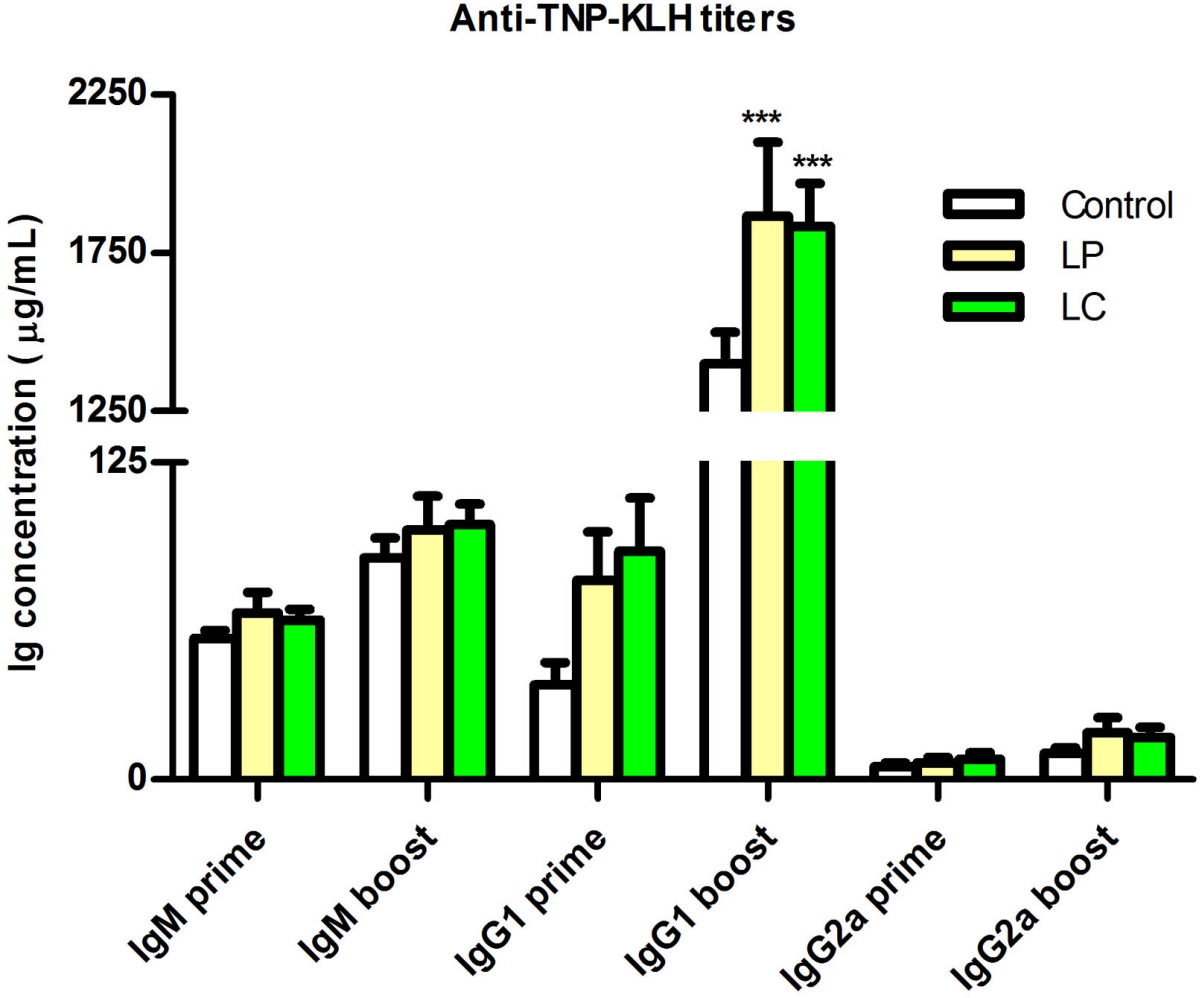
**Supplementation of *L. plantarum* and *L. casei* increased specific anti-TNP-KLH antibody responses of *Ercc1^−/Δ7^* mice**. Mean TNP-specific IgM, IgG1, and IgG2a concentrations in serum were determined by ELISA, 7 days after primary immunization (prime, age 9 weeks), or 7 days after booster immunization (boost, age 13 weeks). Data represent the mean + SEM of 6–12 animals per group. ***p < 0.001. LP, *L. plantarum* WCFS1; LC, *L. casei* BL23.

To test whether changes in immune cell distribution also impact immune competence, we analyzed the B cell response of *Ercc1^−/Δ7^* mice to the T cell-dependent antigen TNP-KLH. Specific anti-TNP-KLH Ig titers of the three tested isotype classes (IgM, IgG1, IgG2a) after primary and booster immunization were consistently higher after *L. plantarum* and *L. casei* supplementation (Figure 9). In particular, IgG1 titers after booster immunization increased in both *L. plantarum*- and *L. casei*-supplemented mice compared with control-treated mice (*p* < 0.001).

From these findings, we conclude that *L. plantarum* and *L. casei* enhance T cell-dependent B cell responses in *Ercc1^−/Δ7^* mice.

## Discussion

The effects of bacterial supplementations on the intestinal barrier and cellular parameters of immunity were studied in fast aging *Ercc1^−/Δ7^* mice. We observed that the mucus layer in the colon declines with age and that bacterial supplementation may prevent or exacerbate the age-related decline in the mucus layer, dependent on the specific bacterial strain. Additionally, we demonstrated a marked difference in the response to bacterial supplementations between *Ercc1^−/Δ7^* mice and WT mice. Finally, supplementation with *L. casei* BL23 profoundly changed the distribution of immune cells and supplementation with *L. plantarum* WCFS1 or *L. casei* BL23 improved immune competence in *Ercc1^−/Δ7^* mice.

Recently, we showed the age-related decline in mucus barrier of C57Bl/6 mice as well (Sovran et al., unpublished data). Importantly, we report that the mucus barrier declines with age, in aged C57Bl/6 and *Ercc1^−/Δ7^* mice (Figure 1). This finding adds another age-related phenotype to the wide spectrum of age-related phenotypes observed in *Ercc1^−/Δ7^* mice (40). Moreover, we report that the age-related decline in mucus barrier can be modulated by bacterial supplementation. *L. plantarum* prevented the decline in mucus barrier. *L. plantarum* is able to bind to mucus with a mannose-specific adhesin, which is described as a potential probiotic feature (41). In total, *L. plantarum* harbors four mucus-binding proteins (42). Based on the improved spatial compartmentalization of bacteria after *L. plantarum* supplementation, we postulate that *L. plantarum* adheres to the mucus. In addition, we found that *L. plantarum* supplementation tended to decrease the abundance of *Akkermansia muciniphila* (Figure 3), which is known as a mucus degrader (43). Thus, it would be conceivable that mucus degradation is decreased after *L. plantarum* supplementation. By contrast, *B. breve* is known as a mucus degrader (44), and could, therefore, be directly responsible for the decrease in mucus thickness in the colon of *B. breve*-treated mice. Interestingly, several pathways involved in protein folding and the UPR were upregulated after *L. casei* and *B. breve* supplementation (Data Sheet 2 in the Supplementary Material). A high demand for synthesis of secretory proteins (such as mucins) induces ER stress, which in turn induces the UPR (45). The close proximity of bacteria to the epithelium in *L. casei-* and *B. breve*-treated mice might induce a high demand for mucin production and secretion, leading to induction of ER stress and UPR. There is indeed evidence that defects in MUC2 mucin and a subsequent defective mucus layer lead to ER stress and UPR (46).

Microbiota profiling showed that only few microbial species are slightly altered by bacterial supplementation (Figures 2 and 3). Therefore, most of the observed effects in the mucus barrier and immune system may be directly linked to the supplementation of each of the bacterial strains.

We found that the different bacterial strains elicited characteristically different responses in gene regulation in the colon (Figure 4). *L. plantarum* is known for its moderately pro-inflammatory profile, and relatively high IL-10 induction, when tested in human PBMC cultures (28, 47). In line with these studies, several upstream regulators predicted to be activated after *L. plantarum* supplementation included the inflammatory cytokines IFN-γ, IL-1β, IL-4, and TNF. The association between increased activation of inflammatory cytokines and the improved integrity of the colon after *L. plantarum* supplementation raises the possibility that it might be beneficial to locally increase inflammatory cytokine levels. This suggestion is corroborated by the absence of activation of these inflammatory cytokines after *L. casei* or *B. breve* supplementation, which did not improve or exacerbate the age-related decline in mucus integrity. A “tonic” level of constitutive TLR activation by commensal bacteria was previously shown to be crucial in the recovery from DSS-induced epithelial damage due to the role of NF-κB in epithelial repair processes (48). This notion that “physiological pro-inflammatory signals” is required for intestinal homeostasis is also supported by studies using epithelium-specific iκB kinase-γ (or NEMO) ablation in mice. These mice develop spontaneous colitis due to the failure of NF-κB to induce epithelial repair and steady-state production of innate effector mechanisms in the intestine (49). TLR2 signaling has been implicated in tight junction regulation *in vivo* and *in vitro* (13). Thus, it is possible that aged mice have sub-optimal level of TLR stimulation in the intestine to promote innate barrier defenses and that this is enhanced by *L. plantarum*, but not by *L. casei* and *B. breve*.

Remarkably, none of the significantly regulated genes were directly linked to mucus production. However, while performing Upstream Regulator Analysis, growth factors, such as EGF, IGF1, and EGR1, were predicted to be activated after *L. plantarum* supplementation. Together, these findings may indicate that mucus production by goblet cells is not directly enhanced, but is part of general epithelial integrity, supported by a number of growth factors.

Because many regulated genes involved immune-related genes, we additionally analyzed the makeup of the immune system after bacterial supplementation. Whereas supplementation with *B. breve* exacerbated the age-related decline in mucus barrier in colon, it did not cause any changes in mucosal or systemic immunity (Figures 5–8). Oppositely, *L. casei* supplementation caused various signs of inflammation, such as Ly6C^hi^ monocyte and neutrophil influx and production in spleen and BM, respectively. These inflammatory signs were coincided with the general decrease in B cell frequencies (also in the BM) and double-positive thymocytes. There is evidence that neutrophils in the BM are primed by microbial ligands (50). The effects of microbiota-derived signals on priming B and T cells in the BM have not been previously described. Our study suggests an, up to now, unknown link between microbiota, intestinal barrier, and B and T cell precursors. Specific precursor stages (i.e., small resting pre-B cells) were significantly decreased after *L. casei* supplementation, and to a lesser extent after *L. plantarum* supplementation. In the case of *L. plantarum* supplementation, we suggest that improved intestinal barrier function might alter circulating microbiota-derived products, such as peptidoglycan (PGN) and lipopolysaccharide (LPS). For instance, hematopoietic stem cells are damaged after chronic exposure to LPS (51). Interestingly, the decrease in small resting pre-B cells after *L. casei* supplementation (and to lesser extent by *L. plantarum*) was the only finding that could be reproduced in WT mice supplemented with these bacterial strains (Supplementary Figure 4 in Data Sheet 1). This may indicate that the effect of *L. casei* and *L. plantarum* supplementation on B cell development is independent of age.

A previous study showed lifespan extension after *B. animalis* supplementation (21). Therefore, we performed a lifespan study for *L. plantarum* and *L. casei*, which indicated that neither of them is shortening or extending lifespan (Supplementary Figure 5 in Data Sheet 1).

Surprisingly, anti-TNP-KLH IgG1 titers in serum increased not only after *L. plantarum* but also after *L. casei* supplementation (Figure 9). This increase suggests that a demise in B cell development and B cell distribution does not necessarily translate into impaired B cell function. Previously, it has been shown that antigen-specific antibody titers can be enhanced by probiotic supplementation in aged mice (20), but data on B cell development are lacking.

The effects of the candidate probiotic strains were pronounced on the mucus barrier in the colon of *Ercc1^−/Δ7^* mice compared with WT mice. It has been shown in previous studies that strains, such as *L. casei* and *B. breve*, have beneficial effects on immunological parameters and intestinal barrier function in young mice (29–31). In our hands, *L. casei* and *B. breve* had no effect on mucus barrier or systemic immunity in young WT mice (except for the above-discussed finding on B cell development). A severe deteriorating effect, however, was observed on the mucus barrier or systemic immunity in *Ercc1^−/Δ7^* mice. These findings highlight the need for caution in translating beneficial effects of probiotics observed in young animals or humans to the elderly.

Our study has a number of limitations. We observed remarkable changes in the mucus layer, but could not pinpoint a single gene that is directly linked to the mucus layer. Furthermore, we did not include commercially available probiotic bacterial strains, such as *Lactobacillus rhamnosus* GG, or a non-probiotic bacterial strain. Nevertheless, our study reveals a previously unknown effect of age on the mucus barrier. We also show that it is possible to modulate this age-related decline in the mucus barrier by supplementation of bacterial strains, with coinciding effects on systemic immunity. More research is warranted to elucidate the interplay between bacteria, the aged gut epithelium, and the immune system.

Our data provide evidence that a comprehensive analysis of the intestinal barrier and immunity are needed in order to evaluate how bacterial supplementation contributes to the restoration of the age-related decline in intestinal barrier. A positive finding was that probiotic strains, such as *L. plantarum*, might contribute to maintenance of intestinal integrity by preventing age-related deterioration of the colonic mucus layer.

## Author Contributions

AB, BS, JHH, WV, PV, JW, PL, CN, RH, and HS conceived the study. AB, BS, FH, BM, CB, JAH, VM, CP, and MB performed the experiments. AB wrote the manuscript. BS, FH, BM, CB, JAH, JHH, WV, PV, JW, PL, CN, RH, and HS contributed to the revisions of the draft manuscripts.

## Conflict of Interest Statement

The authors declare that the research was conducted in the absence of any commercial or financial relationships that could be construed as a potential conflict of interest. The reviewer SS and handling Editor declared their shared affiliation, and the handling Editor states that the process nevertheless met the standards of a fair and objective review.

## Supplementary Material

Data Sheet 1

Supplementary Table 1 **Used antibodies in flow cytometry**.

Supplementary Table 2 **Top-10 biological processes upregulated (as determined with GSEA) by bacterial supplementations in proximal colon of *Ercc1^−/Δ7^* mice treated with *L. plantarum* WCFS1 (LP), *L. casei* BL23 (LC), or *B. breve* DSM20213 (BB)**.

Supplementary Table 3 **Top-10 biological processes downregulated (as determined with GSEA) by bacterial supplementations in proximal colon of *Ercc1^−/Δ7^* mice treated with *L. plantarum* WCFS1 (LP), *L. casei* BL23 (LC), or *B. breve* DSM20213 (BB)**.

Supplementary Figure 1 **Distribution of B cells and T cells in Peyer’s patches and mesenteric lymph nodes not changed upon bacterial supplementation in *Ercc1^+/+^* mice**. **(A/B)** Mean frequencies were determined by flow cytometry. B cells were defined as CD19^+^, T cells were defined as CD3^+^. **(C)** Mean frequencies of CD3^+^CD4^+^CD8^−^FoxP3^+^ regulatory T (Treg) cells in MLN. Data represent the mean + SEM from four to six animals per group. LP, *L. plantarum* WCFS1; LC, *L. casei* BL23; BB, *B. breve* DSM20213.

Supplementary Figure 2 **Increased relative spleen weight after *L. casei* supplementation of *Ercc1*−*/Δ7* mice**. **(A)** Spleen weights relative to body weight. Data represent mean spleen weights + SEM of four to six animals per group. **(B)** Flow cytometric analysis of splenic Th17 cells. CD3^+^CD4^+^CD8^−^ cells were gated for RORγt and FSC (forward scatter).

Supplementary Figure 3 **Bacterial supplementation of *Ercc1^+/+^* mice did not change splenic parameters**. **(A)** Mean frequencies of Treg cells in spleen. **(B–D)** Mean frequencies of Ly6C^hi^ monocytes, neutrophils, and CD3^+^CD4^+^CD8^−^Rorγt^+^ Th17 cells were determined by flow cytometry. **(E)** Mean concentration of IL-17A production by splenocytes stimulated with ConA for 4 days, as determined by Cytometric Bead Array. **(F)** Mean proliferating T cells (Ki-67^+^) in splenocyte culture stimulated with ConA for 4 days, as determined by flow cytometry. Data represent the mean + SEM from four to six animals per group. LP, *L. plantarum* WCFS1; LC, *L. casei* BL23; BB, *B. breve* DSM20213.

Supplementary Figure 4 ***L. casei* supplementation altered B cell development in bone marrow of *Ercc1^+/+^* mice**. **(A–E)** Mean frequencies in bone marrow (BM) were determined by flow cytometry. Granulocyte–monocyte precursors (GMP) were defined as Lin^−^CD117^hi^CD11c^−^CD135^−^CD16/32^+^, neutrophils as CD11b^+^Ly6G^+^, monocytes as Ly6C^hi^CD31^−^, B-lineage cells as CD19^+^CD45R^+^, and small resting pre-B cells as sIgκ/λ^−^cIgM^+^CD2^+^. (**F**) Mean absolute numbers were determined by cell counts and flow cytometry. Double-positive (DP) cells were defined as CD3^−^CD4^+^CD8^+^. Data represent the mean + SEM from four to six animals per group. **p*<0.05. LP, *L. plantarum* WCFS1; LC, *L. casei* BL23; BB, *B. breve* DSM20213.

Supplementary Figure 5 **Bacterial supplementations did not change lifespan of *Ercc1^−/Δ7^* mice**. Data represent 11–12 animals per group (with an additional 6 animals per group censored at 16 weeks). LP, *L. plantarum* WCFS1; LC, *L. casei* BL23.

Data Sheet 2 **Total file with differentially expressed genes after bacterial supplementations**.
