# Supplementary material for: Supplementation with Lactobacillus plantarum WCFS1 Prevents Decline of Mucus Barrier in Colon of Accelerated Aging Ercc1−/Δ7 Mice
Source: Front Immunol. 2016 Oct 7;7:408. doi: 10.3389/fimmu.2016.00408 (PMC5054004; doi:10.3389/fimmu.2016.00408)
Supplement: Data Sheet 1 [file Data_Sheet_1.DOCX]

**Supplementary data**

**Supplementary Table 1. Used antibodies in flow cytometry.**

| Target | Format | Clone | Company |
| --- | --- | --- | --- |
| CD2 | PE | RM2-5 | BD |
| CD3e | APC-Efluor780  FITC*  PerCP-Cy5.5 | 17A2  145-2C11  145-2C11 | Ebioscience  BD  BD |
| CD4 | APC-H7  FITC | GK1.5  H129.19 | BD  BD |
| CD8a | FITC  PE  V450 | 53-6.7 | BD |
| CD11b | APC-Cy7  PE-Cy7 | M1/70 | BD  Ebioscience |
| CD11c | PE-Cy7 | N418 | Ebioscience |
| CD16/32 | APC-Cy7  Purified | 2.4G2 | BD |
| CD19 | APC-Efluor780  FITC*  PerCP-Cy5.5 | 1D3 | Ebioscience |
| CD25 | APC  PE-Cy7 | 3C7  PC61.5 | BD  Ebioscience |
| CD31 | APC | 390 | Ebioscience |
| CD45R/B220 | BV421  FITC* | RA3-6B2 | BD |
| CD68 | FITC | FA-11 | BioLegend |
| CD117 | BV421 | 2B8 | BioLegend |
| CD135 | APC | A2F10.1 | BD |
| FcεRIα | FITC* | MAR-1 | Ebioscience |
| FoxP3 | PE | FJK-16s | Ebioscience |
| IgD | PE  PerCP-Efluor710 | 11.26.2ca  11-26c | BD  Ebioscience |
| Igκ | FITC | 187.1 | BD |
| Igλ | FITC | R26-46 | BD |
| IgM | APC  Efluor450 | II/41 | Ebioscience |
| Ki-67 | PE-Cy7 | SolA15 | Ebioscience |
| Ly6C | AF488  PerCP-Cy5.5 | ER-MP20  HK1.4 | AbD Serotec  Ebioscience |
| Ly6G | BV421  PE | 1A8 | BD |
| Ly6C+Ly6G (GR-1) | FITC* | RB6-8C5 | BD |
| NK1.1 | FITC* | PK136 | Ebioscience |
| RORγt | AF647 | Q31-378 | BD |
| TER-119 | FITC* | TER-119 | BD |

*Included in lineage cocktail for GMP staining.

**Supplementary Table 2. Top-10 biological processes upregulated (as determined with GSEA) by bacterial supplementations in proximal colon of *Ercc1^-/Δ7^* mice treated with *L. plantarum* WCFS1 (LP), *L. casei* BL23 (LC), or *B. breve* DSM20213 (BB).**

| **Nr** | **Pathway** | **NES** | **p-value** | **FDR q-value** |
| --- | --- | --- | --- | --- |
| 1 LP | Malaria (KEGG) | 2.15 | 0.00 | 0.03 |
| 2 LP | Fanconi pathway (NCI) | 2.06 | 0.00 | 0.05 |
| 3 LP | Glycogen metabolism (WIP) | 2.05 | 0.00 | 0.04 |
| 4 LP | ATRBRCA pathway (BIOC) | 1.97 | 0.00 | 0.08 |
| 5 LP | Type II Interferon signaling (WIP) | 1.95 | 0.00 | 0.09 |
| 6 LP | VIP Pathway (BIOC) | 1.94 | 0.00 | 0.08 |
| 7 LP | ARF6 trafficking pathway (NCI) | 1.92 | 0.00 | 0.09 |
| 8 LP | Statin pathway (WIP) | 1.91 | 0.00 | 0.09 |
| 9 LP | Fanconi anemia pathway (KEGG) | 1.85 | 0.00 | 0.14 |
| 10 LP | IL8/CXCR1 pathway (NCI) | 1.83 | 0.00 | 0.15 |
|  |  |  |  |  |
| 1 LC | Mitotic G1-G1 S Phases (REACT) | 1.89 | 0.00 | 0.08 |
| 2 LC | Unfolded protein response (REACT) | 1.90 | 0.00 | 0.10 |
| 3 LC | S Phase (REACT) | 1.83 | 0.00 | 0.12 |
| 4 LC | DNA replication (KEGG) | 1.91 | 0.00 | 0.12 |
| 5 LC | NOD-like receptor signaling pathway (KEGG) | 1.80 | 0.00 | 0.13 |
| 6 LC | Cone pathway (NCI) | 1.81 | 0.00 | 0.13 |
| 7 LC | DNA replication (WIP) | 1.84 | 0.00 | 0.13 |
| 8 LC | Synthesis of DNA (REACT) | 1.78 | 0.00 | 0.14 |
| 9 LC | Glycosphingolipid biosynthesis - lacto (KEGG) | 1.73 | 0.01 | 0.14 |
| 10 LC | G1 S transition (REACT) | 1.78 | 0.00 | 0.14 |
|  |  |  |  |  |
| 1 BB | Citrate (TCA) cycle (KEGG) | 2.00 | 0.00 | 0.07 |
| 2 BB | Prefoldin mediated transfer of substrate to CCT TRIC (REACT) | 1.88 | 0.00 | 0.10 |
| 3 BB | Glycolysis gluconeogenesis (KEGG) | 1.89 | 0.00 | 0.11 |
| 4 BB | Pyruvate metabolism and citric acid (TCA) cycle (REACT) | 1.92 | 0.00 | 0.11 |
| 5 BB | Protein folding (REACT) | 1.75 | 0.00 | 0.12 |
| 6 BB | Fructose and mannose metabolism (KEGG) | 1.82 | 0.00 | 0.12 |
| 7 BB | TCA cycle (WIP) | 1.74 | 0.01 | 0.13 |
| 8 BB | Chaperone-mediated protein folding (REACT) | 1.76 | 0.00 | 0.13 |
| 9 BB | Valine, leucine and isoleucine degradation (KEGG) | 1.77 | 0.00 | 0.13 |
| 10 BB | Butanoate metabolism (KEGG) | 1.72 | 0.01 | 0.14 |

Gene sets significantly regulated (p<0.05, FDR<0.2) by bacterial supplementations compared with control were determined by gene set enrichment analysis (GSEA). Gene sets involved in immunity are highlighted in orange. Gene sets involved in growth and cell cycle are highlighted in blue. NES = normalized enrichment score.

**Supplementary Table 3. Top-10 biological processes downregulated (as determined with GSEA) by bacterial supplementations in proximal colon of *Ercc1^-/Δ7^* mice treated with *L. plantarum* WCFS1 (LP), *L. casei* BL23 (LC), or *B. breve* DSM20213 (BB).**

| **Nr.** | **Name** | **Size** | **ES** | **NES** | **p-value** | **FDR q-value** |
| --- | --- | --- | --- | --- | --- | --- |
| 1 LP | GPCRs (WIP) | 163 | -0.47 | -2.12 | 0.00 | 0.00 |
| 2 LP | Odorant GPCRs (WIP) | 216 | -0.42 | -1.92 | 0.00 | 0.04 |
| 3 LP | Olfactory signaling pathway (REACT) | 44 | -0.53 | -1.89 | 0.00 | 0.04 |
|  |  |  |  |  |  |  |
| 1 BB | IL2 STAT5 pathway (NCI) | 30 | -0.58 | -2.03 | 0.00 | 0.05 |
| 2 BB | Immunoregulatory interactions between lymphoid/non-lymphoid cells (REACT) | 35 | -0.53 | -1.94 | 0.00 | 0.05 |
| 3 BB | Angiopoietin receptor pathway (NCI) | 47 | -0.50 | -1.91 | 0.00 | 0.05 |
| 4 BB | Activation of the pre-replicative complex (REACT) | 21 | -0.61 | -1.91 | 0.00 | 0.05 |
| 5 BB | BARD1 pathway (NCI) | 27 | -0.56 | -1.89 | 0.00 | 0.05 |
| 6 BB | Type II Interferon signaling (WIP) | 30 | -0.56 | -1.97 | 0.00 | 0.05 |
| 7 BB | GPVI-mediated activation cascade (REACT) | 19 | -0.62 | -1.94 | 0.00 | 0.06 |
| 8 BB | Generation of second messenger molecules (REACT) | 16 | -0.64 | -1.87 | 0.00 | 0.06 |
| 9 BB | IL4 2 pathway (NCI) | 57 | -0.48 | -1.97 | 0.00 | 0.07 |
| 10 BB | IL6 7 pathway (NCI) | 45 | -0.47 | -1.81 | 0.00 | 0.07 |

Gene sets significantly regulated (p<0.05, FDR<0.2) by bacterial supplementations compared with control were determined by gene set enrichment analysis (GSEA). Gene sets involved in immunity are highlighted in orange. Gene sets involved in growth and cell cycle are highlighted in blue. NES = normalized enrichment score.

**
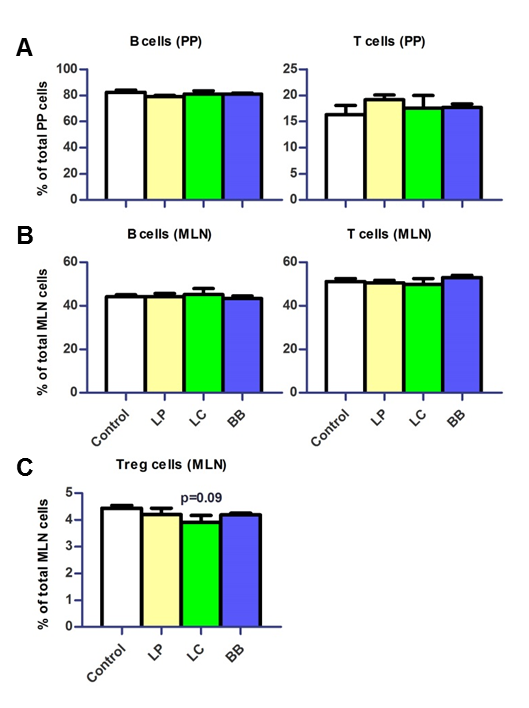
**

**Supplementary Figure 1. Distribution of B cells and T cells in Peyer’s patches and mesenteric lymph nodes not changed upon bacterial supplementation in *Ercc1^+/+^* mice.** A/B) Mean frequencies were determined by flow cytometry. B cells were defined as CD19^+^, T cells were defined as CD3^+^. C) Mean frequencies of CD3^+^CD4^+^CD8^-^FoxP3^+^ regulatory T (Treg) cells in MLN. Data represent the mean + S.E.M. from 4-6 animals per group. LP = *L. plantarum* WCFS1; LC = *L. casei* BL23; BB = *B. breve* DSM20213.

**
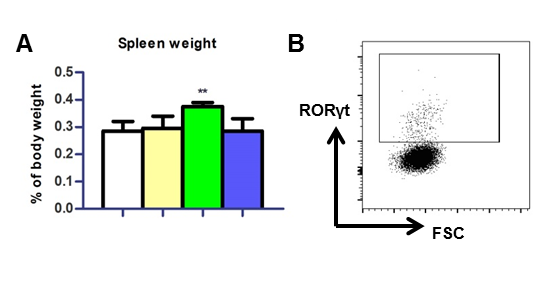
**

**Supplementary Figure 2. Increased relative spleen weight after *L. casei* supplementation of *Ercc1^-/Δ7^* mice.** A) Spleen weights relative to body weight. Data represent mean spleen weights + S.E.M of 4-6 animals per group. B) Flow cytometric analysis of splenic Th17 cells. CD3^+^CD4^+^CD8^-^ cells were gated for RORγt and FSC (forward scatter).

**
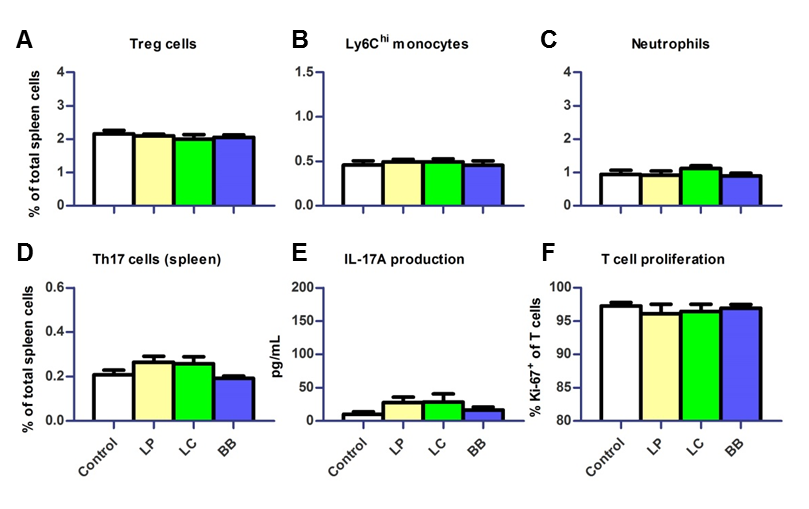
**

**Supplementary Figure 3. Bacterial supplementation of *Ercc1^+/+^* mice did not change splenic parameters.** A) Mean frequencies of Treg cells in spleen. B-D) Mean frequencies of Ly6C^hi^ monocytes, neutrophils, and CD3^+^CD4^+^CD8^-^Rorγt^+^ Th17 cells were determined by flow cytometry. E) Mean concentration of IL-17A production by splenocytes stimulated with ConA for four days, as determined by Cytometric Bead Array. F) Mean proliferating T cells (Ki-67^+^) in splenocyte culture stimulated with ConA for four days, as determined by flow cytometry. Data represent the mean + S.E.M. from 4-6 animals per group. LP = *L. plantarum* WCFS1; LC = *L. casei* BL23; BB = *B. breve* DSM20213.


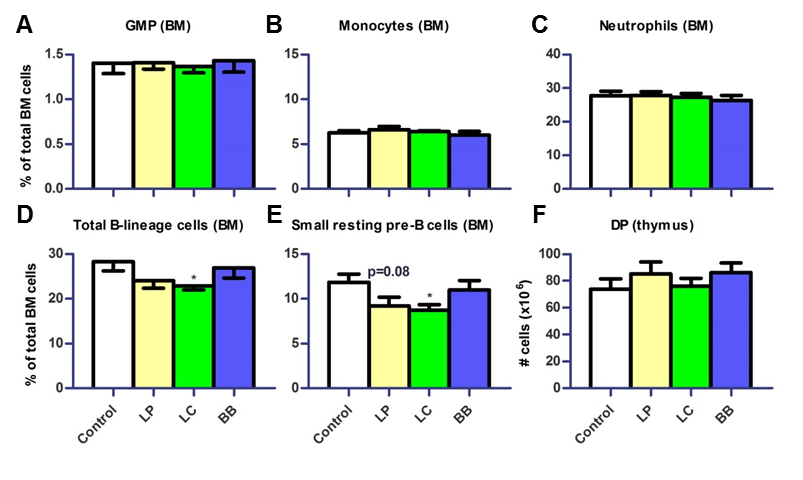


**Supplementary Figure 4. *L. casei* supplementation altered B cell development in bone marrow of *Ercc1^+/+^* mice.** A-E) Mean frequencies in bone marrow (BM) were determined by flow cytometry. Granulocyte-monocyte precursors (GMP) were defined as Lin^-^CD117^hi^CD11c^-^CD135^-^CD16/32^+^, neutrophils as CD11b^+^Ly6G^+^, monocytes as Ly6C^hi^CD31^-^, B-lineage cells as CD19^+^CD45R^+^, and small resting pre-B cells as sIgκ/λ^-^cIgM^+^CD2^+^. F) Mean absolute numbers were determined by cell counts and flow cytometry. Double positive (DP) cells were defined as CD3^-^CD4^+^CD8^+^. Data represent the mean + S.E.M. from 4-6 animals per group. *=p<0.05. LP = *L. plantarum* WCFS1; LC = *L. casei* BL23; BB = *B. breve* DSM20213.


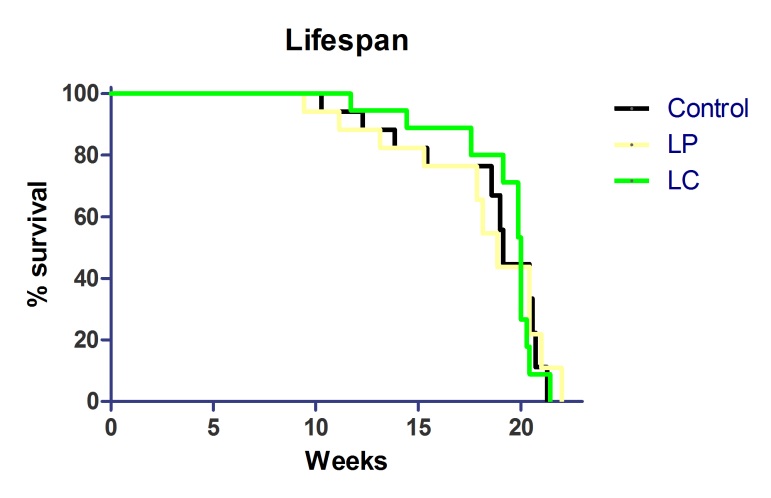


**Supplementary Figure 5. Bacterial supplementations did not change lifespan of *Ercc1^-/Δ7^* mice.** Data represent 11-12 animals per group (with an additional 6 animals per group censored at 16 weeks). LP = *L. plantarum* WCFS1; LC = *L. casei* BL23.
